# Acknowledgment to the Reviewers of *Pharmacy* in 2022

**DOI:** 10.3390/pharmacy11010020

**Published:** 2023-01-20

**Authors:** Pharmacy Editorial Office

**Affiliations:** MDPI AG, St. Alban-Anlage 66, 4052 Basel, Switzerland

High-quality academic publishing is built on rigorous peer review. *Pharmacy* was able to uphold its high standards for published papers due to the outstanding efforts of our reviewers. Thanks to the efforts of our reviewers in 2022, the median time to first decision was 20 days and the median time to publication was 42 days. Regardless of whether the articles they examined were ultimately published, the editors would like to express their appreciation and thank the following reviewers for the time and dedication that they have shown *Pharmacy*:
Abdullah AleneziKaren KierAbhijit PakhareKari L. OlsonAbhishek JuluriKashish MehraAdam DaragóKatarzyna DettlaffAdriana MatosKathleen MazorAfonso M. CavacoKathryn SteadmanAgnieszka SkowronKellie J. GoodletAhmed Mohamed Fahmy YousefKen CorAkshaya BhagavathulaKenneth Lee McCallAleda ChenKerry MansellAleksandra Karczmarska-WódzkaKevin CoulouresAlex N. IsaacsKevin FujiAlina CernasevKevin T. BainAlison ClappKevin T. FujiAllen ShekKim An NguyenAmelia Ramon-LopezKim C. ColeyAmrita SarkarKiriaki KeramitsoglouAmy M. TiemeierKitamura MineakiAmy Theresa PageKristin JankeAna Isabel PlácidoKuan-Hsuan ChenAna PerisinKwang Choon YeeAna TeixeiraKyle Kar Hou TanAna TomasKyle MelinAndrás FittlerKylee FunkAndré SilvaL. Douglas RiedAndrea HiltonLana MinshewAndrea ManfrinLaura CocoAndries KosterLaura Jane SahmAnestis KarakatsanisLaura KnockelAngela M. CocomanLauren LinesAnna BiernasiukLezley-Anne HannaAnthony OlsonLin ZhuAntonio GidaroLinda V. GraudinsAntonio VallanoLisa KroonArit Esio UdohLone HolstArmando Silva-AlmodóvarLong Chiau MingArtur OwczarekLoreta KubilieneAshim MalhotraLorinda AndersonBarry BleskeLouise HughesBeatriz Rodríguez-RocaLusi ZhangBeenish ChaudhryM. Chandra SekarBelen Alonso OrtizMagdalena IorgaBernadette FloodMagdalena Jastrzębska-WięsekBetty ExintarisMagdalena Krajewska-WłodarczykBeverly FitzpatrickMagdalena PizońBiagio RaponeMagdalena Waszyk-NowaczykBianca E. ŐszMahima SinghBoohwi HongMaja Ortner HadžiabdićBorja Herrero De La ParteMajid Mufaqam Syed-AbdulBorut BožičMamoon AldeyabBrian PiperManuel MadrazoBruce SunderlandMarc SturgillCaoimhe ClerkinMarcello CasertanoCarla Maria Batista Ferreira PiresMaree SimpsonCarla PiresMargarida Espírito-SantoCarmen De KockMaría Guadalupe Frías-De-LeónCarolyn M. RichardsonMarie BarnardCaterina PalleriaMariola DrozdCathrin HaukMarion Joy SparkChan-Young KwonMarion SlackCherlie Magny-NormilusMarios SpanakisCheryl CroppMarissa OstroffChiao Xin LimMariusz DuplagaChing-Yi ChangMaroof AlamChis Adriana AureliaMarta HernándezChris GilletteMarta Waliszewska-ProsółChristian CavéMarwa Rashad Rashad SalemChristoph StallmannMary Beth O'ConnellChristopher J. DestacheMatthew WitryChristopher OwensMeghan JeffersClark KebodeauxMelany P. PuglisiClaudia CristianoMelody RyanClaudiu MorgovanMicah HataCody PeerMichael A. BeazelyConxita MestresMichael CeulemansCristina Belén GarcíaMichael DonahoeCristina GalvanMichael RouseCristina Rivera-PicónMichael W. NagyCristoforo PomaraMichelle SahrDaisy VolmerMickael L. D. DerocheDalia AlmaghaslahMiguel Ángel Navas-MartínDana A. BrownMiguel Castelo-BrancoDanguolė RugytėMirko ManchiaDaniel MaloneMirko ProsenDániel NemesMojtaba VaismoradiDaniel Ricardo DelgadoMotohiro OkadaDaniela PierannunzioMuhammad Hammad ButtDarko ModunMumtaz AnwarDavid BrightNancy Monroy-JaramilloDavid H. KrelingNaomi SteenhofDavid WrightNardine NakhlaDebarati DasGuptaNatalia Burgos AlonsoDeeb EidNatalie DiPietro MagerDelia Mirela TitNatalie R. GadboisDerar H. Abdel-QaderNatalya A. ShnayderDerek P. EvansNicole M. GattasDiane M. DolezelNikola StefanovićDobromira ShopovaNikolaos A. ChrysanthakopoulosDoris RusicNilesh PatelEdoabasi U. McGeeOlaf KrauseEduardo Gutiérrez-AbejónOlajide O. FadareEdward P. HofferOoi Guat SeeElisa ZavattaroOsamu KumanoElisabetta FerraraOuti SalminenElizabeth UnniPatrícia PiresElizabeth VafiadakiPaul GallagherEllen Michelle SchellhasePaula L. GrubbEmilien JeannotPaulina StehlikEnrique Sáez-AlvarezPeter Bai JamesEnrique Seoane-VazquezPeter IlicEric BravermanPetra A. ThürmannEricka L. Breden CrousePhil AyersErin HolmesPietro SpataroErshuai ZhangPing ZouEun-Jung KimPiotr PoznańskiEwa TalarekPrawej AnsariFabio MonzaniPriya MartinFatima AroojRagini SinghFernando MendesRainer LeonhartFilipe PrazeresRalph BaergenForoud ShahbaziRaquel Fernandez-GarciaFrancesca GoriniRaúl Tárraga MínguezFulgencio Sánchez VeraRavi PatelGabriela Valdés-RamírezRebecca H. StoneGali NavehRenee RobinsonGalina NikolovaRicardo VardascaGeeta HitchRichard H. Parrish IIGeorg BolligRichard KeersGeorge E. MacKinnon IIIRichard ParrishGeorges AdunlinRieko TakehiraGeorgiana NitulescuRobby MarkwartGeorgios MarinosRodolfo MascheroniGina MooreRoger HarrisonGiulio Di GravioRoman KrálikGiuseppe PonziniRomána ZelkóGonzalo Emiliano Aranda-AbreuRosa M. Cárdaba-GarcíaGregory S. WellmanRosemary KilleenGuendalina ZuccariRuth BreezeGuilhermina Lobato MirandaSaad SalemGuillaume HacheSabine VoglerGyöngyvér SoósSabrina Anne JacobH. M. Ayala S. HerathSandra BenavidesHaider QasimSantosh GeorgeHaishu LinSara WettergreenHans De LoofSarah BillupsHege SletvoldSaval KhanalHelen PergantouSeth T. HousmanHelena BiancuzziShamima KhanHuasheng TongShigeo YamamuraHumberto Javier Rodríguez HernándezSimon Bheki KhozaIbolya CsecsSimona BungauIbolya FülöpSlaven FalamićIlaria Tocco TussardiSofia MattssonIrene Cortés-PérezStefanie FerreriIrina Magdalena DumitruStephanie Y. CrawfordIva MucaloStephen R. TomlinIvan BindoffStevie VeachJ. Lucas ReddingerSungyong ChoiJack E. FinchamSunil S. KarhadkarJacqueline E. McLaughlinSusanne KaaeJagannath MuzumdarSwapna YellankiJan D. HirschTaiwo AremuJan SaevelsTanushree DangiJane E. KrauseTanvee ThakurJane E. TomnayTanya G. SinghJane PortlockTao LanJarred PrudencioTao WangJason GuyTao ZhangJason PerpelkinTeodora Alexa-StratulatJean AmiralTeresa MadureiraJean MoonThomas MawhinneyJeanine P. AbronsTiffany Champagne-LangabeerJean-Venable GoodeTimothy E. WeltyJeff TaylorTimothy NguyenJenny ScottTiziana GuzzoJessica SnowdenTomas TesarJessica ThompsonTora HammarJesús Molina-MulaTram CatJian-Ye ZhangTristan T. TimbrookJoan-Francesc Fondevila-GascónUgochi O. OguJoanne FullerUnn Sollid ManskowJoanne OlsonUrve PaaverJoão Gonçalves-PereiraUyen Minh LeJoão Manuel Graça FradeVanessa MinerviniJóhannes ReynissonVassilios PapadopoulosJohn C. HaydenVenkatakrishnan RengarajanJohn HawboldtVictoria TeamJon P. WietholterVilius SavickasJordan BallouVincent ChanJosé Enrique Moral-GarcíaVincent J. WilleyJosé María León-RubioVishalkumar G. ShelatJosipa BukićVjekoslav PeitlJuan SerraWei-Min ChuJudita KinkorováWenchen WuJumpei SaitoWerner CordierJung-Woo BaeYameng LiJustin ColeYoonJung LeeKah Kheng GohZachary S. ClaytonKai Zhen YapZubin AustinKarel AllegaertZulfan Zazuli


